# “GET-UP” study rationale and protocol: a cluster randomised controlled trial to evaluate the effects of reduced sitting on toddlers’ cognitive development

**DOI:** 10.1186/s12887-016-0723-6

**Published:** 2016-11-09

**Authors:** Rute Santos, Dylan P. Cliff, Steven J. Howard, Sanne L. Veldman, Ian M. Wright, Eduarda Sousa-Sá, João R. Pereira, Anthony D. Okely

**Affiliations:** 1 Early Start Research Institute, School of Education, Faculty of Social Sciences, University of Wollongong, Northfields Avenue, Wollongong, NSW 2522 Australia; 2 Research Centre in Physical Activity, Health and Leisure, Faculty of Sport, University of Porto, Porto, Portugal; 3 Illawarra Health and Medical Research Institute, University of Wollongong, Wollongong, Australia

**Keywords:** Sedentary behaviour, Physical activity, Childcare, Executive function

## Abstract

**Background:**

The educational and cognitive differences associated with low socioeconomic status begin early in life and tend to persist throughout life. Coupled with the finding that levels of sedentary time are negatively associated with cognitive development, and time spent active tends to be lower in disadvantaged circumstances, this highlights the need for interventions that reduce the amount of time children spend sitting and sedentary during childcare. The proposed study aims to assess the effects of reducing sitting time during Early Childhood Education and Care (ECEC) services on cognitive development in toddlers from low socio-economic families.

**Methods/Design:**

We will implement a 12-months 2-arm parallel group cluster randomised controlled trial (RCT) with Australian toddlers, aged 12 to 26 months at baseline. Educators from the ECEC services allocated to the intervention group will receive professional development on how to reduce sitting time while children attend ECEC. Participants’ cognitive development will be assessed as a primary outcome, at baseline and post-intervention, using the cognitive sub-test from the Bayley Scales of Infant and Toddler Development.

**Discussion:**

This trial has the potential to inform programs and policies designed to optimize developmental and health outcomes in toddlers, specifically in those from disadvantaged backgrounds.

**Trial registration:**

Australian New Zealand Clinical Trials Registry: ACTRN12616000471482, 11/04/2016, retrospectively registered.

**Electronic supplementary material:**

The online version of this article (doi:10.1186/s12887-016-0723-6) contains supplementary material, which is available to authorized users.

## Background

The early years are critical in setting the trajectory of an individual’s life. Healthy child development up to the age of 5 years provides the basis for a prosperous and sustainable society [1]. The material and psychosocial context of poverty adversely affects multiple aspects of development in children [2, 3] and developmental deficits associated with poverty start before birth and can be detected as early as infancy [4, 5]. The best available knowledge on the basic principles of neuroscience indicate that providing supportive conditions for early childhood development is more effective and less costly than attempting to address the consequences of early adversity later [3]. Indeed, early life experiences from birth to school entry are essential to build strong neurodevelopmental trajectories crucial for long-term social and occupational functioning [6].

Specifically, the first 5 years of life are important for the development of executive functions (EFs). EFs are often conceived as higher-order cognitive control processes, which consist of three inter-related functions. The first, working memory, is involved in “holding information in mind and working with it” [7], which is important for everything from understanding language to mental mathematics and reasoning. Central to working memory is the activation of information via attention. Inhibition, by contrast, involves “inhibiting (or suppressing) attention [or action] to other things in the environment (distracters) so you can stay focused on what you want” [7]. Lastly, cognitive flexibility involves an ability “to shift mental sets or see something from different perspectives” [7]. Together, these cognitive control processes enable us to perform the full range of everyday and extra-ordinary tasks that are essential for adaptive human function.

The cognitive control provided by EFs is essential for the behavioural, emotional and social controls that contribute to positive life trajectories. That is, ample research has indicated a link between EFs and school readiness, academic success, social and emotional development, criminality and employment success [7]. Moreover, in the largest prospective study of self-regulation conducted thus far conducted so far (1000 children who were followed from birth to age 32), it was reported that childhood self-control (at ages 3 to 11) predicted better physical health, less substance dependence, higher personal finances and less criminal offending outcomes in adulthood, following a gradient of self-regulation, even after adjusting the analysis for IQ, sex, and social class while growing up [8]. More than fixed trajectories of self-regulatory development, this study also demonstrated the malleability of self-regulation over time, as well as improved trajectories when this occurred.

Early EFs are also considered the “*biological foundation for school readiness”* [7, 9] and a better predictor of academic achievement than IQ [10, 11]. It has been shown that EF skills at the age of 4 years provided children with an immediate advantage in the school learning environment and a head start in maths and reading that was maintained through to the age of 7 years [12]. Fitzpatrick et al. [13] reported that early working memory (at the age of 29 and 41 months) predicted later (at 74 months of age) classroom engagement, number knowledge and receptive vocabulary, independent of sex, non-verbal intellectual skills and socio-economic status.

Cognitive development is affected by several socio-economic and environmental factors. Disadvantaged children usually experience less cognitive stimulation and enrichment, watch more television, attend lower quality childcare, and have poorer diets [14]. These children may also be exposed to stressful environments and receive less warmth, stability and support from their families [14]. The cumulative effect of these risk factors during a sensitive period of brain expansion and growth can compromise neurocognitive development [5, 15]. Indeed, the educational and cognitive differences associated with low socioeconomic status begin early in life and tend to persist throughout life [11, 16]. For example, Lipina et al. [4] reported that infants (aged 6 to 14 months) from disadvantaged families had, on average, less developed working memory and inhibitory control abilities compared to those from non-disadvantaged families. Evans and Schamberg [17] also reported a prospective association between the duration of childhood poverty and adult working memory, an association that appeared to be explained in part by elevated chronic stress during childhood. Therefore, targeting EF, in early childhood in order to improve school readiness and later academic success seems crucial [18] and may constitute an important tool to reduce the academic achievement gap between more and less-advantaged children.

In a 2011 review, Diamond and Lee [18] identified six types of interventions that successfully improve EFs in children aged 4 to 12 years: computerised training; a hybrid of computer and noncomputer games; classroom curricula; adds-ons to classroom curricula; aerobic exercise and martial arts and mindfulness practices. These interventions have revealed that: (i) children with lower EF (including those from socially disadvantaged backgrounds) benefit the most from any EF intervention; (ii) EF training in one area seems to transfer to other EFs, but this transfer is limited; and (iii) executive demands need to be repeated and continually incremented [7, 18]. All of these interventions have two important features in common: first, “*the programs tend to reduce stress in the classroom, cultivate pride, joy and self-confidence, and foster social bonding*”; and second, “*they do not expect children to sit still for long—such expectations are not developmentally appropriate, increase teacher-student tensions, and lead some children to dread school and/or to be wrongly labels as having Attention Deficit and Hyperactivity Disorder (ADHD)*” [18].

Although these types of interventions have been successful from 4 years of age, recent evidence recommends that they should start even earlier than the pre-school period—i.e. between 1 and 2 years of age—to attenuate the impact of disadvantage on the children’s cognitive development [19, 20]. New ideas and new approaches are therefore necessary [21].

It has been long recognised that PA is positively associated with cognitive performance in school-aged children (aged 4 to 18 years) [22] and across the lifespan [23]. In infants, passive cycling for 2 months during the first year of life resulted in positive motor (body control balance, grasping), adaptive (hand–eye coordination) and language gains (communication by facial expression, sounds, vocalizations, and babble) compared to controls [24].

Early childhood is also a critical period to establish long-term sedentary and physical activity (PA) behaviours [25]. Sedentary behaviour (SB) refers to waking activities that do not increase energy expenditure substantially above the resting level, while in a sitting or lying position [26]. It has been suggested that SB should be explicitly measured either for surveillance purposes or research studies instead of being defined as lack of PA [27] as sedentariness and PA are two independent and not mutually exclusive behaviours with potentially different effects on development and health outcomes [28–31].

Studies assessing PA and SB levels in infants and toddlers are scarce [32], particularly using objective measures of PA and SB. Recently, Cardon et al. [32] summarised the literature and reported that PA rates are low, infants and toddlers spend a large proportion of their time being sedentary. Television viewing is already common at these ages. For example, in the US it is estimated that 17 % of 0–11 month old and 48 % of 12–23 months old children exceed the American Academy of Pediatrics guidelines (<2 h/day) [33] and that 40 % of 3-months old babies and 90 % of 2 years old toddlers watch TV regularly [34]. Some studies have shown that PA levels of children aged 0 to 5 years are typically low and SB high [35–37]. In a meta-analysis of objectively measured PA, Bornesteins et al [38] reported that pre-schoolers spend on average only 5.5 % of their waking time in moderate-to vigorous-intensity PA. Australian pre-school children also are characterized as highly inactive, with only 5 % meeting the current national recommendations of 3 h/day of PA [39]. ECEC settings may be extremely inactive and sedentary environments [40] with children spending only 15 % of their childcare day engaged in PA [41] and up to 80 % of their day in sedentary activities [42]. This suggests that young children may not be provided with the opportunity to move in ways that are natural and developmentally appropriate even at ECEC centres. This is particularly concerning, given that several studies have reported beneficial associations between PA and several health indicators and developmental aspects in early childhood [43, 44] and also the deleterious health effects of excessive SB [44].

High levels of SB (particularly screen time) during early childhood have shown to be associated with higher adiposity [44, 45], higher blood pressure [46], less bone accrual [47], attention problems [48] language development [49], psychosocial health [44, 50] and cognitive development [44, 51]. However, a recent systematic review showed that different types of SB may have different associations with cognitive development during early childhood: reading/being read to was positively associated with cognitive development in contrast to screen-based activities [51]. Moreover, a longitudinal study has documented that the time spent watching television under the age of five was negatively related to cognitive development, short-term memory and academic achievement 1 to 3 years later [52].

Evidence suggests that there are several neurological pathways that may explain the adverse links between sitting and cognitive development [30]. These include pathways involving the birth of new neurons (neurogenesis) that occurs mainly in the hippocampus, a critical area in the brain for learning and memory processes. Prolonged sitting may promote increased activation of stress systems through its rapid effect on metabolic and inflammatory pathways, and chronic stress activation may slow the rate of neurogenesis. Sitting may also negatively affect synaptic plasticity (creation of new or strengthening of existing synaptic connections during learning) through pathways involving insulin and adipose-driven leptin. Additionally, Growth Factors (BDNF, VEGF, IGF-1) play multiple roles in the survival and maturation of new neurons, which are critical for neurogenesis, synaptic plasticity and angiogenesis (growth of new blood vessels); over-abundance of insulin caused by prolonged sitting may suppress systemic IGF-1 signaling that promotes neuronal growth and repair. Since insulin-sensitivity is disrupted shortly after breaking up prolonged sitting, IGF-1 signaling may also be affected. It is known that PA improves cerebral blood flow due to increases in artery diameter, the sprouting of new capillaries from existing vessels, and the bioavailability of endogenous nitric oxide. It has been hypothesized that prolonged sitting decreases nitric oxide and may promote endothelium dysfunction [30]. Despite this evidence, the influence of PA and SB on cognitive development in toddlers remains poorly understood [44].

In this context, and in an attempt to respond to recent calls on innovative approaches and fresh thinking on how to improve cognitive development in disadvantaged children [21], the primary aim of this study is to assess the effects of reducing sitting time during ECEC on cognitive development, in a cluster randomised controlled trial with toddlers from low socio-economic families. It is hypothesized that at 12-month follow-up, toddlers in centres allocated to the intervention group will have improved their cognitive development by 0.5 SD more than toddlers in ECEC centres randomly allocated to the control group. The secondary aims are to examine the effects of reducing sitting time on toddler’s cardiovascular health and bone density.

## Methods

### Study design

We will implement a 12-months 2-arm parallel group cluster randomised controlled trial. Cluster randomization was chosen for practical reasons and to prevent contamination. The selection of the ECEC services (clusters) will be based on the ECEC’ postcode using the Australian Socio-economic Indexes for Areas 2011 (SEIFA-Index of Relative Socio-Economic Disadvantage) [53]. We will consider the centres belonging in the first and second quartile of this Index (extreme-low and moderate-low socio-economic status) in the Illawarra Region, NSW, Australia. Eight services will be randomly allocated to the intervention group and eight to the control group.

### Study context

Early childhood education and care for toddlers is not compulsory in Australia. Australian children in their toddler years are enrolled in Child Care by their families’ choice. In Australia, 22.2 % of children aged 0–1 year and 54.1 % of children aged 2–3 years attend formal care at least once a week (http://www.abs.gov.au).

There are four types of formal care in Australia being Long Day Care, Occasional Care, Family Day Care and In Home Care; these services must be registered and approved by the Department of Education and Communities to operate. The Government in each state regulates Child Care Services. The following standards of practice in Child Care are adhered to: The National Childcare Accreditation Council Standards; Education and Care Services National Regulations; Children Education and Services National Law Act 2010; the Early Years Learning Framework; the United Nations Conventions on the Rights of the Child and the Australian Early Childhood Code of Ethics. In Australia the ratio educators/childcare workers per child is 1:4 for 0 to 1 year-olds and 1:8 for 2 to 3 year-olds (www.acecqa.gov.au).

ECEC services in Australia are income tested. Families earning under $152,147/annum with one child are able to apply for Child Care Benefit and Child Care Rebate from the Government. The income threshold goes up with each additional child in the family. However, the eligibility for this benefit is also dependant on the child’s immunisation status. The average fee for a Long Day Care service is $102/day (www.abs.com.au).

Some Long Day Care Services provide all the necessary meals of (breakfast, morning and afternoon tea and lunch) whilst others may provide only a snack and parents are required to bring lunch and other snacks. In the option of the latter, these services charge a lower fee than those whom provide meals.

For the purpose of this study data will be collected only in Long Day Care Services in NSW.

### Ethics statement

The University of Wollongong’s Human Research Ethics Committee approved the study (HE15/236) and this RCT was registered in the Australian and New Zealand Clinical Trials Registry (ACTRN12616000471482, 11/04/2016, retrospectively registered) (see Additional file 1: Table S1 for trial registration data details). Informed written consents will be obtained from the Educators and children’s parents or guardians. The study will be conducted according to the Helsinki Declaration for Human Studies [54].

### Participants and protocol

#### Inclusion criteria

This RCT will comprise 16 ECEC services from Illawarra region in NSW, each of which must have at least one class of at least 20 toddlers, from a low- to medium- socioeconomic background [53]. All apparently-healthy toddlers aged 12- to 26-months at baseline will be eligible to participate if they attend the ECEC service at least twice per week. We expect to recruit 18 participants per childcare services. Children will be considered ineligible if they have a learning or physical disability, born very preterm (<29 weeks of gestation) or have a diagnosed medical or psychological condition that would affect the results of this study.

#### Recruitment

After determining the eligible ECEC services, we will perform a simple randomisation using a computerised sequence generation to determine the order in which eligible ECEC services will be invited to participate in this study. Invitations will be performed by email and by phone, requesting a face-to-face meeting with service Director to outline the aims and procedures required for this RCT.

If a selected ECEC service declines participating in the study, an additional phone call and face-to-face meeting will be made to the next eligible service on the list until 16 childcare services agree to participate in this RCT.

All toddlers enrolled in each class/ECEC centre will be invited to participate in the study. We will send an invitation letter and an information sheet outlining the aims and procedures of the study to the toddler’s parents or guardians. While no exclusion criteria will be applied for participation in the study, to prevent any discrimination, for analysis and reporting only toddlers eligible according to the aforementioned inclusion criteria will participate in the study.

### Intervention

The intervention is based on Bandura’s Social Cognitive Theory [55], which has been used extensively in behaviour change interventions. Social Cognitive Theory posits that behaviour is learned, modified and sustained through the interplay of personal, behavioural and environmental factors. The intervention will focus on these factors and how they influence sitting behaviours. All components of the intervention have been designed to address the four key learning processes suggested by Bandura [56] to enhance behaviour change (*attention, retention, production*, *motivation).* The intervention has also been designed to target Social Cognitive Theory mediators, such as educator self-efficacy [57].

The intervention has been designed in response to formative research conducted by the authors within similar ECEC services in disadvantaged locations. In 2013, 12 focus groups were held with educators from 11 services. Educators were provided with data on how much time children in their service spent sitting and asked to identify by how much they would like to reduce this proportion of time. The most common reduction was 50 % of the time currently spent sitting. Based on the proportions presented above, this would result in reducing a “typical” day (50 % of the time in childcare spent sitting) to 25 % of the time spent sitting. Educators were then asked to complete a daily routine log for their service, which described the modifications that would take place to allow the halving of time spent sitting. Researchers then provided further ideas. The ideas provided by educators largely focused on changes to practices and modifications to the physical environment. Included in these ideas were ways to ensure no bouts of sitting exceeded 15 min. These example schedules will be used by each of the intervention ECEC services to design their own daily schedule to reduce total sitting time by 50 % and to reduce bouts of sitting to <15 min.

**Fig. 1:**
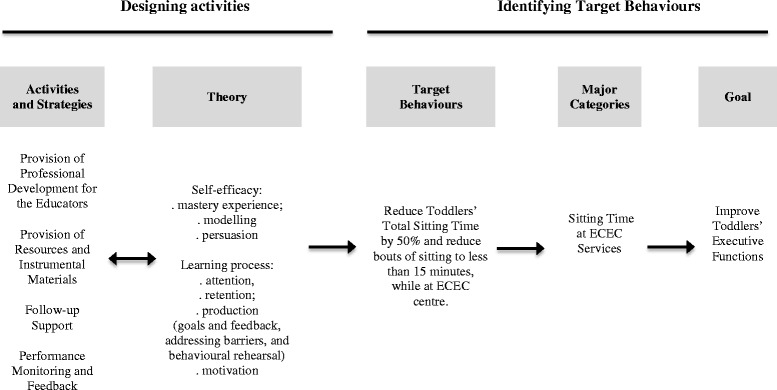
Effects of reduced sitting time on toddlers’ cognitive development: a cluster randomized controlled trial. Intervention mapping. ECEC = Early Childhood Education and Care

**Fig. 2:**
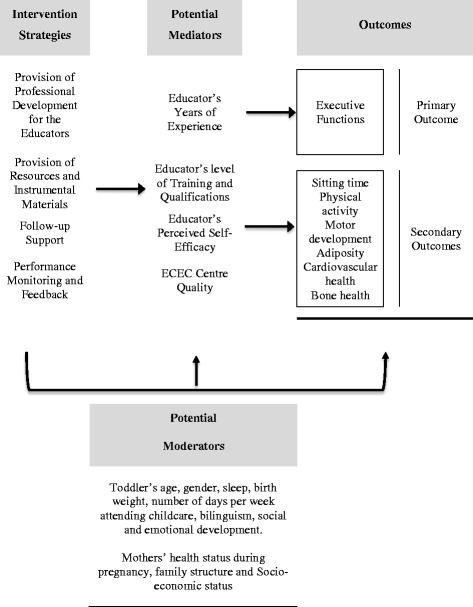
Effects of reduced sitting time on toddlers’ cognitive development: a cluster randomized controlled trial. Potential mediators and moderators. ECEC = Early Childhood Education and Care

We mapped the intervention step-by step as proposed by Robinson and colleagues [58]. We began by identifying the target behaviours and then worked backwards to define activities and strategies and potential mediators. (see Figs. 1 and 2). To attain our goal of improving toddler’s executive functions we will implement four main strategies, which are based on the principles of the Bandura’s Social Cognitive Theory: (i) Professional Development for Educators, (ii) Provision of Resources and Instrumental Materials (ii) Follow-up Support and (iv) Performance Monitoring and Feedback (as described in detail in Table 1), to reduce toddler’s total sitting time by 50 % and to reduce bouts of sitting to less than 15 min, while toddlers are in the ECEC centre.

**Table 1:** Effects of reduced sitting time on toddlers’ cognitive development: a cluster randomized controlled trial. Intervention strategies and activities and corresponding principles of Social Cognitive Theory

Activities and strategies	Description	Principle of Social Cognitive Theory
Provision of Professional Development for the Educators	Educators will attend a 6 h professional development workshop. The workshop will begin by introducing the rationale and aims of the study (1 h).	Self-efficacy:
		. mastery experience;
	Educators will then be asked to think about ways to (i) modify routine activities to reduce the total amount of sitting time and reduce bouts of sitting to less than 15 min and (ii) to change indoor and outdoor environment to reduce the total amount of sitting time and the length of the sitting bouts. (1 h).	. modelling
		. persuasion
	After this activity, the educators will be able to rehearsal some of the proposed activities in our laboratory (2 h and 30 min).	Learning process:
	Finally, educators will be given further ideas on how to reduce toddlers’ total sitting time and reduce bouts of sitting to less than 15 min. Perceived barriers for the implementation of the program and possible solutions to overcome these barriers will be discussed (1.5 h).	. attention,
		. retention;
		. production (goals and feedback, addressing barriers, and behavioural rehearsal)
	We will aim to train all educators from each centre on the same day to ensure standardization of content delivery.	
Provision of Resources and Instrumental Materials	After the professional development workshop we will provide educators with supporting written materials with the rationale, aims and strategies/activities to reduce toddler’s sitting time. We will provide them with posters to be displayed in their classroom as a reminder of the need to reduce sitting time. A video demonstrating the proposed activities and desired routine changes will also be provided.	Learning process:
		. retention;
		. motivation
		Self-efficacy:
		. modelling
Follow-up Support	During the intervention period the educators will receive monthly on-site visits from the research team, to revise key activities and behavioural strategies to reduce toddlers’ total sitting time and to follow up on the activities that are being undertaken.	Self-efficacy:
		. mastery experience;
		. persuasion
	Three months and six months after the start of the intervention educators will attend an interactive online webinar to follow-up on the intervention, to share ideas and perceived barriers of the intervention implementation and to address possible solutions to overcome those barriers.	Learning process:
		. retention;
		. production (goals and feedback, addressing barriers)
	During the intervention period educators will also receive regular emails and telephone calls.	
		. motivation
Performance Monitoring and Feedback	During the monthly visits the research staff will collect objective information on the total sitting time and sitting bouts (by accelerometry) in a random small sample of toddlers (10 %) to monitor the implementation of the intervention. This performance will be delivered to the educators, providing feedback on the intervention implementation.	Self-efficacy:
		. mastery experience;
		. persuasion
		Learning process:
		. retention;
		. production (goals and feedback, addressing barriers)
		. motivation

Table 2 describes in detail the main activities to be proposed to the educators attending the professional development workshop. As explained in Table 1, after the professional development workshop further activities on how to reduce total sitting time and sitting bouts can be added to this list. The initial list of activities presented in Table 2 includes changes in routine activities, changes in the indoor environment and changes in the outdoor environment.

**Table 2:** Effects of reduced sitting time on toddlers’ cognitive development: a cluster randomized controlled trial. Activities to be proposed to educators

Activities	Description of the activities for the educators
Routine activities	
Action time story	Role-playing stories. For example, when telling a story about a horse, every time the children hear the word horse (or the horse character’s name) they have to jump up and pretend to be a horse for 5 s. Repeat this throughout the story. The group can be broken up into smaller groups of frogs, crocodiles, horses etc. Where each group has to stand up at only their prompt. This activity works for Executive Function abilities—working memory—as children have to remember what animal they are and it decreases sedentary behaviour.
Stand on a dot or a hoop to gather children as a group.	Asking children to stand on a dot or in a hoop would replace sitting on the floor to gather them as a group. This activity would still give the control and structure of gathering and focusing the children, but reducing sitting time. The children can also be gathered and asked to hold hands while waiting for the others to arrive. In doing this children are making their own barrier and focusing their attention on the group.
Musical painting table	When the children are painting at a standing table, put on some music, after 2 min stop the music and the children have to put down their paintbrushes/pencils and change spots around the table. Then the music starts and they start painting again. The painting can be done on a big sheet of paper on the table, or a sheet on the table, or on individual pieces of paper.
Apply sunscreen with the toddlers standing	Have all children standing while applying the sunscreen.
Indoor environment	
Locating play/learning spaces/areas near the wall	By locating play/learning spaces/areas near the wall and leaving the centre of the room with an open space, children have increased opportunities to move freely from one place to other.
Standing table	Remove the chairs away from the table so that the children can be standing while painting, doing puzzles or other activities.
Move pencils/brushes away form the painting table	Place the pencils and brushes in a separate table so that children can move from one table to other to change pencils/brushes.
Move bins away from the tables during meal times	Place the bins away from the tables during meal times, so that children need to walk to bin after the meals.
Outdoor environment	
Remove chairs and tables from the outdoor space	Remove all equipment that promotes sitting (chairs and tables) from the outdoor space
Use a tree in the yard as an easel	Attach paper to the tree and do chalk rubbings of the bark from the tree. Paper would be at standing height.
Painting along the fence with an old sheet	In warmer days, paint along the fence with an old sheet with the children standing.
Provide equipment that does not promote sitting (ex. balls)	During outdoors free play provide toddlers with equipment that does not promote sitting (ex. balls).

### Control Group

The Control Group will continue with their usual program and will receive the intervention training and materials at the end of follow-up assessments.

### Assessment of the Outcomes

**Fig. 3:**
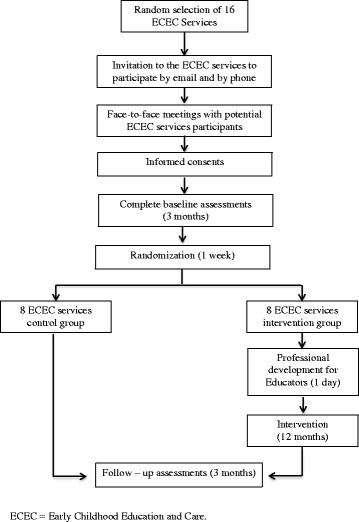
Effects of reduced sitting time on toddlers’ cognitive development: a cluster randomized controlled trial. Participants Timeline

Data collection will occur at baseline (before randomization) and at the end of the intervention (Fig. 3 Participants Timeline). For those children who have turned 3 at follow-up, an additional four executive function tasks will be collected as described below. Trained research assistants blinded to group allocation will collect all data.

#### Primary Outcome

##### Cognitive development

Cognitive development and executive functions will be assessed using the cognitive sub-test from the Bayley Scales of Infant and Toddler Development—Third edition (Bayley-III) [59]. This sub-test assesses sensorimotor development, exploration and manipulation, object relatedness, concept formation, memory, and problem solving. The Cognitive subset of the Bayley scales will be applied and scored according to standard procedures described in the Bayley-III manual for the age of the child at the starting point. For those children who have turned 3 at follow-up, an additional four executive function from the Early Years Toolbox will be used to ensure assessments are comprehensive. These iPad-based tasks assess the three core aspects of EF, and have been developmental and psychometrically evaluated with large samples of preschool-aged children [60]. All iPad apps have built-in auditory instructions so the data collectors could ensure the participant understood the instructions, clarified where necessary and remained on task.

The Early Years Toolbox go/no-go task [61] evaluates the ability to inhibit a dominant behavioural response in response to a less frequently presented ‘no-go’ stimulus. In this game, a child is asked to ‘catch the fish’ by tapping on the screen when they appear (‘go’ response) and ‘avoid the sharks’ by not pressing anything when the less-frequent sharks appear (‘no-go’ response). The task is evenly split into three mixed blocks of 25 stimuli, each consisting of 80 % ‘go’ trials, so as not to exceed 1 min for each block and to provide participants with a short break between each block. Animated stimuli (swimming from left to right across the screen) are presented in random order for 1500 ms each, followed by a 1000 ms interval between stimuli. Scores represent the product of proportional go and no-go accuracy.

The Early Years Toolbox Mr. Ant task assesses visual-spatial working memory, or the amount of visual information that concurrently can be activated in the mind. The child is presented with an image of a cartoon ant, which has coloured dots on different spatial locations on his body. Mr Ant, with his coloured dots, is presented for five seconds, followed by a blank screen for four seconds, and then an image of Mr. Ant without any coloured dot appears and the participant indicates the recalled locations by tapping on the recalled locations. In the first level, with a single dot, children must remember a single spatial location. The task proceeds until the earlier of failure on all three trials at a given level of difficulty or level eight (with the location of eight coloured dots to recall). Performance is indexed by a point score, such that each successive level with at least two trials correct receives 1 point, and all correct trials thereafter receive 1/3 of a point.

The third Early Years Toolbox task, a card sorting task, is a measure of cognitive flexibility (the ability to disengage and re-direct attention) [61]. In this task, children are presented with stimuli, one at a time, which vary in shape and colour (red rabbit and a blue boat). Children are asked to sort the shapes into castles (denoted by a blue rabbit and red boat) first by one dimension (colour) and then, after six trials, by another dimension (shape). Successful completion of at least five pre-switch and post-switch trials results in administration of a border version of the task, in which children must flexibly switch between these sorting rules depending on whether the stimulus does or does not have a border. Scores represent the number of correct trials once the initial switch has been made.

Cognitive assessments and EF tests will be conducted individually in a private area and will be scheduled on separate days, where possible, to minimise cognitive fatigue.

#### Secondary Outcomes

##### Sitting time

Total time spent sitting and bouts of sitting during childcare hours will be assessed over a 1 week period using an activPAL accelerometer [62]. The activPAL (PAL technologies, Glasgow) is small (53 x 35 x 7 mm) and lightweight (15 g) and is placed on the front of the upper thigh (using a small hypo-allergenic adhesive gel patch, and covered with a transparent sticky film to secure it) allowing it to measure different postures (eg, sitting, standing). Concurrent and criterion validity of the activPAL for sitting time measures, as well as for interruptions (breaks—defined as the number of transitions recorded from “sit/lie” posture to “stand”) in SB have been established for young children [62–64].

##### Physical activity and sedentary time

Levels of physical activity and sedentary time over a usual week will be measured using Actigraph GT3X+ accelerometers. Actigraphs are small, light and unobtrusive devices worn on a belt around the waist. These accelerometers have established validity and utility in toddlers [65, 66]. These devices can collect very high-frequency raw data (30 Hz), which will be reintegrated and analysed according to best-practice methodologies at the time of analysis. Participants will be asked to wear the accelerometer for 24 h/day over 7 days (except for water activities), and parents will be asked to register in an activity monitor log the times that the accelerometers was removed from the child.

##### Demographics

Demographic and family lifestyle variables will be assessed with a family survey. This survey will include the following variables: parents/caregiver’ age, gender, marital status, Indigenous/Torres Strait Islander origin; child’s gestational age at birth; family structure and family socio-economic status. Family socio-economic status will be assessed by the family postcode address using the Australian Socio-economic Indexes for Areas 2011 (SEIFA-Index of Relative Socio-Economic Disadvantage) [53] and also with a modified version of the Graffar index [67] which includes the highest level of schooling completed, income, main source of income and type of employment (job title). The family survey will also assess parental height and weight and smoking and alcohol habits [68, 69].

##### Sleep

Sleeping patterns will be assessed with the Tayside Children’s Sleep Questionnaire [70, 71]. This 10-item scale evaluates the child’s ability to initiate and maintain sleep. The family survey also asks parents to report their child’s total sleeping time per day. Sleep duration will also be objectively measured with the Actigraph GT3X+ accelerometers.

##### Psychological adjustment

Educators will assess children’s psychological adjustment with the extended version of the Strengths and Difficulties Questionnaire (SDQ). This questionnaire asks about the emotional symptoms, conduct problems, hyperactivity-inattention, peer problems and pro-social behaviour of the child [72, 73].

##### Anthropometrics

We will measure weight, height and waist circumference according to standard procedures [74]. Body height will be measured to the nearest 0.1 cm in bare or stocking feet with the child standing upright against a portable stadiometer (Seca 254 Hamburg, Germany). Body weight was measured to the nearest 0.10 kg, lightly dressed (and without diapers) using a portable electronic weight scale (Seca 254 Hamburg, Germany). Waist circumference will be measured with a non-elastic tape at the top of the iliac crest [75, 76].

##### Blood pressure

Blood pressure will be measured with a digital vital signs monitor using an appropriate size cuff (WelchAllyn PROBP 3400 series, Skaneateles Falls, NY: USA), in a quiet room between 7 and 9 a.m. Two measurements will be taken after 5 and 10 min of rest with the participant in a sitting position, with the arm relaxed and supported so that the cubital fossa is at the level of the heart. Measurements will be taken from the right arm using an appropriate cuff size. A third measurement will be taken if the difference between the previous two measurements was more than 2 mm Hg [77–79].

##### Retinal microvasculature

Changes in the retinal micro-vessels are believed to precede chronic conditions such as heart disease and diabetes [80, 81]. Retinal microvasculature will be assessed by retinal photography, as a direct and non-invasive visualization of the body’s microvasculature. The image of each eye will be recorded using a portable retinal camera (Optomed Smartscope Pro, Finland) according to the manufacturer instructions. The child will be asked to look into the camera and focus on the red dot that will be floating in their visual space. Once the eye is focused and in the right position, a photo will be taken of each eye [82]. Images will be analysed for arterial and venular diameters, arteriovenous ratio and vessel tortuosity using appropriate software.

##### Bone mineral density

Bone mineral density will be assessed using a portable ultrasound bone sonometer (Pediatric Sunlight MiniOmni, BeamMed Ltd., Israel) which non-invasivly measures bone speed of sound (in meters per second). Results and then expressed as age- and gender-matched Z-scores and percentiles. The measurements will be performed on the left leg at the mid-tibia (point between the apex of the medial malleolus and the distal patellar apex), while the participant and the operator are comfortably seated, according to the protocols recommended by the manufacturer (www.beammed.com).

##### Mother’s health during pregnancy

The family survey will assess several variables about the mother’s health during pregnancy that are known to be related with our primary outcome and/or cardiovascular health of the children. We will assess weight gain during pregnancy [83], weeks of gestation [84], type of birth delivery (vaginal delivery, instrumental vaginal delivery or caesarean) [85, 86], singleton and multiparous pregnancy [87], health problems during pregnancy (Hypertension or Pre-eclampsia, Gestational Diabetes, Type II Diabetes, Type I Diabetes, Vitamin D deficiency, Anaemia, Cardiovascular disease, Thyroid dysfunction) [88–92] smoking and alcohol consumption during pregnancy [93], vitamins supplementation and physical activity habits [94].

##### Educators’ demographics and self-efficacy

Educator’ age, gender, level of schooling, qualification and years of experience will be self-reported. Educators will also complete a modified version of the teacher self-efficacy scale of Bandura [95]. The questionnaire will assess instructional self-efficacy; disciplinary self-efficacy and self-efficacy to create a positive childcare climate.

##### Childcare environmental rating

We will rate ECEC centres with the Infant/Toddler Environment Rating Scale-revised edition (ITERS-R). This scale is designed to assess the structural and process quality of early childhood programs. The scale contains 39 items that comprise seven subscales: (i) space and furnishings; (ii) personal care routines; (iii) listening and talking; (iv) activities; (v) interaction; (vi) program structure; and (vii) parents and staff [96].

### Sample Size

#### Sample size and power calculations

We anticipate an effect size of 0.5 for the between-group difference in cognitive development and an intraclass correlation (ICC) of 0.01–0.05. The proposed design has 16 services (8 per group) and 16 children completing per service. Allowing for participation of 18 eligible children with two dropouts per service [10 %] gives a total sample size of 256. This reduces to an effective sample size of 200–254 (with rounding) based on ICCs of 0.05−0.01. The power to detect an effect size of 0.5 with these sample sizes ranges from 0.87–0.96 at an alpha level of 0.05.

### Randomisation and allocation

Research assistants will conduct baseline assessments before randomization. ECEC services will be randomly allocated to either intervention or control condition. We will create a randomization sequence using excel 2011 (Microsoft, Redmond, WA, USA) with a 1:1 allocation using random block sizes of 2, 4 and 6. An independent statistician will conduct this procedure and the data manager will perform the random allocation of services.

### Blinding

Randomization and group allocation will be blinded for the data collectors of this RCT.

### Implementation

Process evaluation will include fidelity of the implementation, consistency of the implementation across ECEC services, and barriers to implementation. To assess fidelity and consistency of the intervention, educators will complete a weekly checklist documenting the activities that were undertaken in the ECEC centre. During the intervention period educators will receive monthly visits from the research team for process evaluation. During these visits we will assess objective information on the sitting time in a small random sample (10 %) of toddlers to monitor the implementation of the intervention. On these occasions we will also assess the educators’ perceived barriers of the intervention implementation with semi-structured interviews. Attendance rates for each child will also be collected to account for the dose of intervention received.

### Data Management

In compliance with the University’s policies, all study-related information will be stored securely at the University, in locked filing cabinets in locked offices, and will be treated as strictly confidential.

Data will be entered directly into existing, secure online or offline databases. All databases are secured by password-protected access systems. For quality control of data entry, another member of the research team will do a random check of data entry quality in 10 % of sample cases. The confidentially and anonymity of the data will be secured through a coding system of the participants. Re-use of the anonymised data will be made available for future projects by arrangement. All participants will also have access to their own results. A data monitoring committee will not be established due to the fact that the interventions poses no risk to the participants.

### Statistical analysis

#### Primary analysis

Analysis of the primary outcome will be conducted using a linear or generalized mixed model in STATA 14.0 (or higher). The mixed model will contain a random effect for time and service nested within group. Degrees of freedom will be altered manually in the code to adjust for the effect of clustering. These established procedures are well documented by Murray [97] and have been used previously by our research institute to analyse a similar study in primary schools [98]. No interim analyses are planned.

#### Secondary analyses

Mixed models will also be used to analyse the differences between treatment and control groups for all continuous secondary outcome variables.

#### Mediation and moderation analyses

Two types of analyses will be conducted to explore the theoretical assumptions of the intervention. First, hypothesised mediators of change in cognitive development (e.g., educator self-efficacy) will be examined using multilevel linear analysis and a product-of-coefficients test appropriate for cluster RCTs. Potential moderators of the intervention effects (e.g., child age and gender) will also be explored using multi-level modelling.

## Discussion

Evidence suggests that early childhood development is critical for the establishment of the foundations for future learning, and social and health outcomes [6, 7] and even small improvements in cognitive development and EF during early childhood “could shift the entire distribution of outcomes in a salutary direction and yield large improvements in health, wealth and crime rate for a nation” [8]. The proposed RCT represents the first study aimed to assess the effects of decreased sitting on cognitive development and EF in toddlers. The results of this study could significantly inform SB and PA guidelines for the early years, as SB experimental studies and studies with toddlers using objectively-measured SB and PA have been identified as critical to enhance the quality of the evidence base [99, 100]. Moreover, research regarding the potential harms of excessive sitting and benefits of PA in toddlers are scarce [32, 44] and a call has been made for further research on cognitive and psychosocial development to inform SB guidelines for the early years due to the lack of evidence in this area [101]. This RCT aims to address this knowledge deficit.

This RCT will also take an innovative approach and use unique methodologies to significantly increase current understanding. In children SBs such as television viewing and overall screen time have been commonly studied and rely on parent-proxy reports; however, they do not represent the total amount of habitual sedentary time. This project will provide new insights on SB and cognitive development in toddlers by: (i) including direct comprehensive and standardized measures of cognitive development for young children; (ii) objectively assessing SB and PA with accelerometry (iii) testing independent associations of SB and cognitive development (e.g. adjusting the analysis for PA levels); (iv) testing mediators of change in cognitive development and moderators of the intervention effects and (v) using a cluster RCT design which is critical for establishing cause and effect relationships and dose-response associations.

Through better understanding of the effects of reducing sitting time on cognitive development and EF in disadvantaged toddlers, this RCT has the potential to inform programs and policies designed to optimize developmental and health outcomes in young children, specifically in those from a disadvantaged backgrounds.

This project has the potential to inform future government policies and programs focused on SB and PA such as updating the National Physical Activity Recommendations for Children 0–5 years [102].

In conclusion, the knowledge generated by this project could be beneficial, nationally and internationally, for: i) parents aiming to optimize developmental outcomes for their children; ii) early childhood educators, clinicians, health care providers, and health promoters, aiming to enhance developmental outcomes in young children through intervention programs; iii) government departments seeking to develop evidence-based guidelines; and iv) researchers seeking to evaluate and translate effective programs to give young children the best start in life.

## Additional file

Additional file 1: Table S1. Effects of reduced sitting time on toddlers’ cognitive development: a cluster randomized controlled trial. Trial registration data. (DOCX 76 kb)

## Abbreviations

ECEC: Early childhood education and care
EF(s): Executive function(s)
PA: Physical activity(ies)
SB: Sedentary behavior(s)
