# Supplementary material for: “GET-UP” study rationale and protocol: a cluster randomised controlled trial to evaluate the effects of reduced sitting on toddlers’ cognitive development
Source: BMC Pediatr. 2016 Nov 9;16:182. doi: 10.1186/s12887-016-0723-6 (PMC5103395; doi:10.1186/s12887-016-0723-6)
Supplement: Additional file 1: Table S1. — Effects of reduced sitting time on toddlers’ cognitive development: a cluster randomized controlled trial. Trial registration data. (DOCX 76 kb) [file 12887_2016_723_MOESM1_ESM.docx]

Table S1 – Table 1 – Effects of reduced sitting time on toddlers’ cognitive development: a cluster randomized controlled trial. Trial registration data.

| Data Category | Information |
| --- | --- |
| Primary registry and trial identifying number | Australian and New Zealand Clinical Trials Registry ACTRN 12616000471482 |
| Date of registration in primary registry | 11/04/2016 |
| Secondary identifying numbers | UTN U1111-1175-8691 |
| Primary source of monetary or material support | Australian Research Council |
| Primary sponsor | Dr Rute Santos |
| Secondary sponsor | Dr Rute Santos [rutes@uow.edu.au] |
| Contact for public queries | Dr Rute Santos [rutes@uow.edu.au] |
| Contact for scientific queries | Dr Rute Santos [rutes@uow.edu.au] |
| Public title | Effects of reduced sitting time on toddlers’ cognitive development: a cluster randomised controlled trial. |
| Scientific title | Effects of reduced sitting time on toddlers’ cognitive development: a cluster randomised controlled trial. |
| Countries of recruitment | Australia |
| Health condition(s) or problem(s) studied | Cognitive development |
| Intervention | Intervention group - 12 month reduced sitting time |
| Key inclusion and exclusion criteria | Age eligibility: 12 to 26 months at baseline |
|  | Inclusion: toddlers from low socio-economic backgrounds attending long day Early Childhood Education and Care services at least twice a week |
| Study type | Interventional |
|  | Allocation: randomized |
|  | Masking: blind |
| Date of first enrolment | 01/03/2016 |
| Target sample size | 256 |
| Recruitment status | Recruiting |
| Primary outcome | Cognitive development |
| Key secondary outcomes | Sitting time |
|  | Physical Activity |
|  | Cardiovascular health |
|  | Bone mineral density |
